# Diseases of the Teeth and Mouth as Causes of Organic Disease

**Published:** 1922-05

**Authors:** 


### DISEASES OF THE TEETH AND MOUTH AS CAUSES OF ORGANIC DISEASE

Much evidence has accumulated in recent years to suggest that dental and oral infections are often causative factors in bringing on various forms of organic disease. Isolated cases of such important conditions as rheumatic fever and various heart affections have been traced by physicians to infected teeth; and, as if to prove their contention, symptoms of these conditions have disappeared or subsided on the removal of the focus of infection in the mouth. Such isolated instances, however, have not been sufficiently numerous to permit of any safe generalization. It has seemed desirable, therefore, to make a study of this subject to determine the degree to which certain organic diseases can be traced to original foci in the mouth. During the last nine months the New York State Dental Society and the Metropolitan Life Insurance Company have co-operated in making such a study among the Industrial policyholders of the Company. Letters of inquiry were sent out to physicians in all cases during this period where the cause of death of the policyholder seemed to indicate the possibility of oral infection as a source, and the physicians were requested to indicate whether dental or oral infection was, in fact, a causative factor in the fatal disease. The results, to date, are very interesting and suggestive.

A total of 774 replies was received to 1,232 letters of inquiry. In 167 or 21.6 per cent. of the cases, the physician stated that infection of the teeth or buccal cavity was present; in 61 or 7.9 per cent. of the 774 cases they stated definitely that they considered the buccal cavity infection as a distinct causative factor to which the disease, which eventually caused death, was a sequel. Thus, out of 43 inquiries with reference to acute articular rheumatism, 14 per cent. were positive as to the buccal cause. In 98 cases of myocarditis, 8 or 8.2 per cent. were reported as positive. In 117 cases of mitral regurgitation, 11 or 9.4 per cent. were so returned. In 144 anemia cases, 10 or 7 per cent. gave mouth infection as the primary cause. In 118 cases of ulcer of the stomach, 9 or 7.2 per cent. were positive; and in 95 cases of infectious endocarditis, 8 or 8.4 per cent. were positive.

In addition to the diseases above mentioned, the replies gave indications that mouth infections frequently cause fatal arthritis deformans, osteomyelitis, septicemia, chronic gastritis and meningitis. We must wait, however, for a larger number of cases in connection with these diseases. The results are negative, so far, for pericarditis and for skin diseases.

These results, while based on too small numbers to be conclusive are, obviously, very suggestive, and justify further inquiry into this subject. The impression of dentists and physicians as to the gravity of mouth infection as a cause of serious organic disease appears to be borne out by these preliminary results. The investigation will be continued until a sufficient number of cases is available to form a basis for definite conclusions as to the importance of mouth infections as known causative factors in fatal cases of several important organic diseases.

A more detailed report will be made by the New York State Dental Society at its annual meeting next May.—*Metropolitan Insurance Bulletin,* March, 1922.

